# Lentivirus-modified hematopoietic stem cell gene therapy for advanced symptomatic juvenile metachromatic leukodystrophy: a long-term follow-up pilot study

**DOI:** 10.1093/procel/pwae037

**Published:** 2024-06-25

**Authors:** Zhao Zhang, Hua Jiang, Li Huang, Sixi Liu, Xiaoya Zhou, Yun Cai, Ming Li, Fei Gao, Xiaoting Liang, Kam-Sze Tsang, Guangfu Chen, Chui-Yan Ma, Yuet-Hung Chai, Hongsheng Liu, Chen Yang, Mo Yang, Xiaoling Zhang, Shuo Han, Xin Du, Ling Chen, Wuh-Liang Hwu, Jiacai Zhuo, Qizhou Lian

**Affiliations:** Cord Blood Bank, Guangzhou Institute of Eugenics and Perinatology, Guangzhou Women and Children’s Medical Center, Guangzhou Medical University, Guangzhou 510623, China; State Key Laboratory of Pharmaceutical Biotechnology, Department of Medicine, The University of Hong Kong, Hong Kong SAR 999077, China; Department of Haematology, Guangzhou Women and Children’s Medical Center, Guangzhou Medical University, Guangzhou 510623, China; Cord Blood Bank, Guangzhou Institute of Eugenics and Perinatology, Guangzhou Women and Children’s Medical Center, Guangzhou Medical University, Guangzhou 510623, China; Department of Pharmacy, Shenzhen Second People’s Hospital, The First Affiliated Hospital of Shenzhen University, Shenzhen University School of Medicine, Shenzhen University, Shenzhen 518060, China; Shenzhen University of Advanced Technology, Key Laboratory of Quantitative Synthetic Biology, Shenzhen Institute of Synthetic Biology, Shenzhen Institutes of Advanced Technology, Chinese Academy of Sciences, Shenzhen 518055, China; Department of Hematology and Oncology, Shenzhen Children’s Hospital, Shenzhen 518026, China; Cord Blood Bank, Guangzhou Institute of Eugenics and Perinatology, Guangzhou Women and Children’s Medical Center, Guangzhou Medical University, Guangzhou 510623, China; Shenzhen University of Advanced Technology, Key Laboratory of Quantitative Synthetic Biology, Shenzhen Institute of Synthetic Biology, Shenzhen Institutes of Advanced Technology, Chinese Academy of Sciences, Shenzhen 518055, China; Department of Haematology, Shenzhen Second People’s Hospital, The First Affiliated Hospital of Shenzhen University, Shenzhen University School of Medicine, Shenzhen University, Shenzhen 518060, China; Department of Haematology, Shenzhen Second People’s Hospital, The First Affiliated Hospital of Shenzhen University, Shenzhen University School of Medicine, Shenzhen University, Shenzhen 518060, China; State Key Laboratory of Pharmaceutical Biotechnology, Department of Medicine, The University of Hong Kong, Hong Kong SAR 999077, China; State Key Laboratory of Pharmaceutical Biotechnology, Department of Medicine, The University of Hong Kong, Hong Kong SAR 999077, China; Department of Anatomical and Cellular Pathology, The Chinese University of Hong Kong, Hong Kong SAR 999077, China; Department of Paediatrics, Shenzhen Second People’s Hospital, The First Affiliated Hospital of Shenzhen University, Shenzhen University School of Medicine, Shenzhen University, Shenzhen 518060, China; Department of Child Neurological Rehabilitation, Maternal & Child Health Hospital, Shenzhen 518000, China; State Key Laboratory of Pharmaceutical Biotechnology, Department of Medicine, The University of Hong Kong, Hong Kong SAR 999077, China; State Key Laboratory of Pharmaceutical Biotechnology, Department of Medicine, The University of Hong Kong, Hong Kong SAR 999077, China; Department of Radiology, Guangzhou Women and Children’s Medical Center, Guangzhou Medical University, Guangzhou 518026, China; Cord Blood Bank, Guangzhou Institute of Eugenics and Perinatology, Guangzhou Women and Children’s Medical Center, Guangzhou Medical University, Guangzhou 510623, China; Shenzhen University of Advanced Technology, Key Laboratory of Quantitative Synthetic Biology, Shenzhen Institute of Synthetic Biology, Shenzhen Institutes of Advanced Technology, Chinese Academy of Sciences, Shenzhen 518055, China; Scientific Research Center, The Seventh Affiliated Hospital, Sun Yat-sen University, Shenzhen 518107, China; Department of Hematology and Oncology, Shenzhen Children’s Hospital, Shenzhen 518026, China; State Key Laboratory of Pharmaceutical Biotechnology, Department of Medicine, The University of Hong Kong, Hong Kong SAR 999077, China; Department of Haematology, Shenzhen Second People’s Hospital, The First Affiliated Hospital of Shenzhen University, Shenzhen University School of Medicine, Shenzhen University, Shenzhen 518060, China; State Key Laboratory of Pharmaceutical Biotechnology, Department of Medicine, The University of Hong Kong, Hong Kong SAR 999077, China; Department of Paediatrics and Medical Genetics, Taiwan University Hospital, Taipei 110024, China; Department of Haematology, Shenzhen Second People’s Hospital, The First Affiliated Hospital of Shenzhen University, Shenzhen University School of Medicine, Shenzhen University, Shenzhen 518060, China; Cord Blood Bank, Guangzhou Institute of Eugenics and Perinatology, Guangzhou Women and Children’s Medical Center, Guangzhou Medical University, Guangzhou 510623, China; State Key Laboratory of Pharmaceutical Biotechnology, Department of Medicine, The University of Hong Kong, Hong Kong SAR 999077, China; Shenzhen University of Advanced Technology, Key Laboratory of Quantitative Synthetic Biology, Shenzhen Institute of Synthetic Biology, Shenzhen Institutes of Advanced Technology, Chinese Academy of Sciences, Shenzhen 518055, China

**Keywords:** HSCGT, advanced symptomatic, metachromatic leukodystrophy, juvenile, patients, safety, efficacy

## Abstract

Metachromatic leukodystrophy (MLD) is an inherited disease caused by a deficiency of the enzyme arylsulfatase A (ARSA). Lentivirus-modified autologous hematopoietic stem cell gene therapy (HSCGT) has recently been approved for clinical use in pre and early symptomatic children with MLD to increase ARSA activity. Unfortunately, this advanced therapy is not available for most patients with MLD who have progressed to more advanced symptomatic stages at diagnosis. Patients with late-onset juvenile MLD typically present with a slower neurological progression of symptoms and represent a significant burden to the economy and healthcare system, whereas those with early onset infantile MLD die within a few years of symptom onset. We conducted a pilot study to determine the safety and benefit of HSCGT in patients with postsymptomatic juvenile MLD and report preliminary results. The safety profile of HSCGT was favorable in this long-term follow-up over 9 years. The most common adverse events (AEs) within 2 months of HSCGT were related to busulfan conditioning, and all AEs resolved. No HSCGT-related AEs and no evidence of distorted hematopoietic differentiation during long-term follow-up for up to 9.6 years. Importantly, to date, patients have maintained remarkably improved ARSA activity with a stable disease state, including increased Functional Independence Measure (FIM) score and decreased magnetic resonance imaging (MRI) lesion score. This long-term follow-up pilot study suggests that HSCGT is safe and provides clinical benefit to patients with postsymptomatic juvenile MLD.

## Introduction

Metachromatic leukodystrophy (MLD) is an inherited genetic lysosomal storage disease caused by dysfunction of arylsulfatase A (ARSA) or its activator prosaposin (PSAP) (Biffi et al., 2008). Reduced ARSA activity leads to the accumulation of sulfatides in the nervous system, resulting in demyelination, neuroinflammation, and neurodegeneration (Shaimardanova et al., 2020). MLD is commonly classified into three clinical subtypes according to the age at first symptoms: late infantile (0–2.5 years), juvenile (2.5–18 years), and adult (after 18 years) (Gieselmann and Krägeloh-Mann, 2010; Schoenmakers et al., 2022). The progression of neurodegeneration in patients with late infantile and juvenile MLD is much more aggressive than that in adult MLD, with progressive loss of motor and neurocognitive function within a few months and death within a few years of symptom onset (Shaimardanova et al., 2020). Treatment of MLD presents many challenges, particularly when most patients are at an advanced symptomatic stage at diagnosis. ARSA enzyme replacement therapy and intracerebral-administered adeno-associated virus gene therapies have shown limited efficacy in halting disease progression for these patients (Shaimardanova et al., 2020). Only patients with juvenile MLD in the presymptomatic stage can benefit from allogeneic hematopoietic stem cell (HSC) transplantation (Biffi et al., 2013; Boucher et al., 2015; Fumagalli et al., 2022; Groeschel et al., 2016; Sessa et al., 2016). Nonetheless, the clinical application of HSC transplantation is limited by the rapid disease progression, a lack of HLA-matched donors, and the risk of graft-versus-host disease (GVHD). Recently, lentivirus-modified hematopoietic stem cell gene therapy (HSCGT) has been developed based on autologous HSC transplantation following *ex vivo* transduction with lentiviral vectors encoding human *ARSA* cDNA. These genetically modified autologous HSCs with high ARSA activity are transfused into MLD patients prior to the symptomatic stage (Biffi et al., 2013; Fumagalli et al., 2022; Sessa et al., 2016). This HSCGT has been shown to effectively preserve cognitive function and motor development in children with presymptomatic MLD and halt demyelination and brain atrophy (Biffi et al., 2013; Fumagalli et al., 2022; Sessa et al., 2016). Nonetheless most patients have progressed to a postsymptomatic stage at diagnosis due to a long diagnostic process and insufficient awareness of MLD, as well as the absence of obvious motor or neurocognitive dysfunction in the presymptomatic stage (Schoenmakers et al., 2022; Shaimardanova et al., 2020). There is no proven effective treatment for these patients whose mobility and cognitive function are largely impaired (Harrington et al., 2019). In contrast to the early-onset forms of infantile MLD, late-onset forms, such as juvenile and adult MLD, usually present with a more insidious manifestation of a wide range of neurological symptoms, providing ample opportunity for potential therapeutic interventions. There is an urgent need to evaluate the safety and efficacy of HSCGT in this real-world population. We undertook a long-term follow-up study to determine whether HSCGT could benefit patients with postsymptomatic juvenile MLD.

## Results

### Patient characteristics

**Figure 1.**
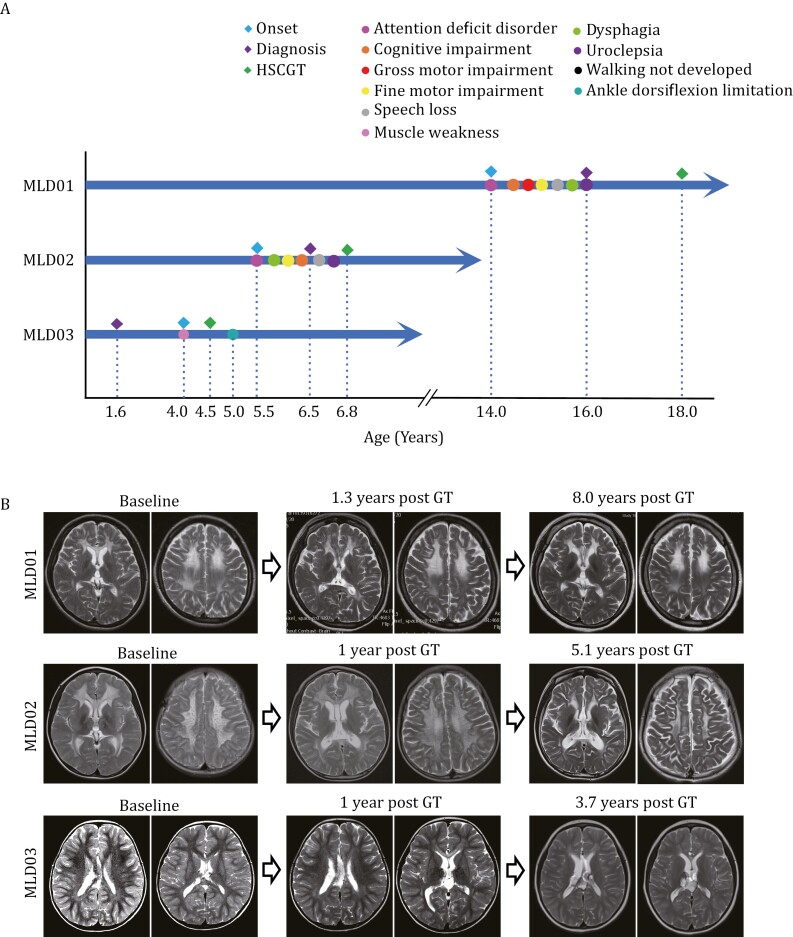
**The disease course and MRI assessment of MLD patients.** (A) Disease course of MLD patients. MLD01 presented with disease onset at the age of 14 years with gradual speech loss, and cognitive, gross, and fine motor impairment prior to diagnosis of MLD at age 16 years. This patient eventually received HSCGT at the age of 18 years when uroclepsia had developed. MLD02 suffered from dysphagia, attention deficit, gross and fine motor impairment at the age of 5.5 years. By the age of 6.5 years, she had developed frequent uroclepsia and was diagnosed with MLD and underwent HSCGT at age 6.8 years. MLD03 was diagnosed with MLD at the age of 1.6 years, discovered an intracranial lipoma at the age of 2.5 years, developed muscle weakness at the age of 4.0 years, and received HSCGT at age 4.5 years. Within 6 months of HSCGT, MLD03 presented with tight Achilles tendons and external rotation of both lower extremities that has been stable to date after one year of HSCGT. (B) Representative brain magnetic resonance imaging (MRI) showing the status of the white matter before (baseline) and after HSCGT at different time points during long-term follow-up and assessment of brain lesions.

Six patients with advanced symptomatic juvenile MLD were assessed for HSCGT eligibility based on the inclusion/exclusion criteria. Three patients were excluded, including one who had progressed to end-stage disease while awaiting transplantation and two who withdrew consent and underwent allogeneic haematopoietic stem cell transplantation (HSCT) from a human leukocyte antigen (HLA)-matched donor. Three young patients were enrolled in this HSGCT pilot study, all of whom were unable to undergo allogeneic HSCT, either due to the absence of an HLA-compatible donor or a high risk of GVHD. Patient MLD01 was diagnosed with attention deficit disorder at the age of 14 years. She was withdrawn from school due to successive disorders including cognitive impairment, gross and fine motor impairment, speech loss, dysphagia, and uroclepsia, all diagnosed before 16 years of age. No extra-neurological symptoms were noted in this patient. There was no abnormality at birth or family history. Attention deficit disorder was also first noted in patient MLD02 at the age of 5.5 years. The patient was then hospitalized for neurodegenerative symptoms including dysphagia, fine motor impairment, and cognitive impairment. After a diagnosis of MLD at the age of 6.5 years, symptoms worsened with aphasia and uroclepsia prior to HSCGT. No extra-neurological symptoms were noted, and no abnormalities were evident at birth or in the family history. Patient MLD03 was diagnosed 1.6 years prior to the onset of the disease following screening based on positive family history in her sibling. Intracranial lipoma was detected as an extra-neurological disorder at the age of 2.5 years. The patient developed symptoms of muscle weakness with signs of white matter lesions at the age of 4 years prior to HSCGT. The course of the disease in these patients was progressive prior to treatment (Fig. 1A). The severity of brain lesions in MLD patients was assessed by MRI and scored using the MRI lesion score system (Eichler et al., 2009). After treatment, MRI showed brain lesions were not progressive and stabilized during the follow-up periods in these patients (Fig. 1B).

### Treatment and safety assessments

**Figure 2.**
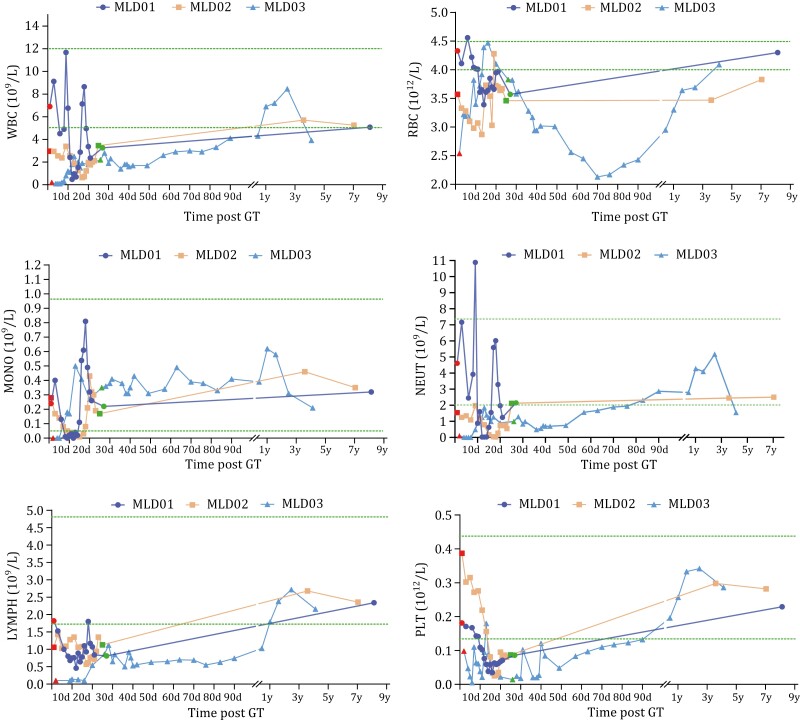
**Hematological monitoring.** Patients’ myeloablation status was monitored before and after discharge. Neutropenia resolved before discharge in all patients. The red dot represents the time of myeloablative treatments, the green dotted line represents the range of normal values, and the green dots represent the last time of hematological monitoring of patients before discharge. For long-term follow-up, the last hematopoietic status monitoring was at the age of 7.9 years for MLD01, 6.9 years for MLD02 and 4.5 years for MLD03. Abbreviations: WBC, white blood cells; MONO, monocytes; NEUT, neutrophils; LYMPH, lymphocytes; RBC, red blood cells; PLT, platelets; d, days; y, years.

Three patients received HSCGT and were followed for 4.5–9.6 years to assess long-term safety and clinical benefit (Table 1, cutoff date 8 May 2024). Safety profiles were analyzed for the primary endpoint based on AEs occurring during both short- and long-term follow-up. No evidence of delayed hematopoietic reconstitution was observed after 2 months of monitoring and all AEs, including neutropenia, resolved with supportive care during short-term follow-up (Tables 1 and S1; Fig. 2). Long-term follow-up to assess the safety of HSCGT treatment continued for 9.6 years in MLD01, 6.9 years in MLD02, and 4.5 years in MLD03. No HSCGT-related AEs were observed (Tables 1 and S1). There was no evidence of impaired or skewed hematopoietic differentiation in long-term peripheral blood monitoring including absolute white blood cells, red blood cells, platelets, lymphocytes, neutrophils, and monocytes (Fig. 2).

**Table 1.** Characteristics of patients.

Variant	MLD01	MLD02	MLD03
Demographics			
Ethnicity	Chinese	Chinese	Chinese
Sex	Female	Female	Female
Age at onsets, years	14.0	5.5	4.0
Age at gene therapy, years	18.1	6.8	4.5
Clinical features			
Clinical subtype	Juvenile	Juvenile	Juvenile
* ARSA* mutation	c.251G > A; c.439delA	c.257G > A; c.827C > T	c.925G > A; c.1237G > A
* PSAP* mutation	None	None	None
Patient conditioning			
Busulfan			
Dose, mg/kg	0.8	1.2	1.1
Total dosage, mg	768.0	432.0	281.6
Duration of neutropenia			
Severe^a^, days	3.0	5.0	5.0
Absolute^b^, days	3.0	2.0	2.0
HSCGT dose, ×10^6^ cells/kg	7.1	10.0	7.8
Duration of follow-up, years	9.6	6.9	4.5
Long-term outcomes			
ARSA activity, nmol/17 h/mg			
Baseline	4.23	3.68	15.70
Last follow-up	85.18	84.62	171.91
FIM score			
Baseline, motor points	36.0	85.0	91.0
Last follow-up, motor points	89.0	55.0	91.0
Baseline, cognition points	6.0	15.0	35.0
Last follow-up, cognition points	24.0	17.0	35.0
Baseline, total score points (level)	42.0 (maximal assistance)	77.0 (moderate assistance)	126 (complete independence)
Last follow-up, total score points (level)	113.0 (modified independence)	78.0 (moderate assistance)	126 (complete independence)
GMFC-MLD			
Baseline, points (level)	4	0	0
Last follow-up, points (level)	0	1	0
MRI lesion score			
Baseline, points (level)	18.0 (moderate)	21.0 (severe)	1.0 (mild)
Last follow-up, points(level)	16.0 (moderate)	14.0 (moderate)	9.0 (moderate)
Laboratory tests			
VCN of CD34^+^			
Baseline, copy/cell	0.0	0.0	0.0
After transduction, copy/cell	0.21	0.79	0.32
VCN of PBMC			
Baseline, copy/cell	0.0	0.0	0.0
Last follow-up, copy/cell	0.16	0.08	0.26

^a^Severe neutropenia refers to an ANC < 500 × 10^6^ neutrophils/L.

^b^Absolute neutropenia was considered an ANC of 0 neutrophils/L.

Abbreviations: ND, not done; PT, post treatment.

### Genomic-integrating efficiency assessment

**Figure 3.**
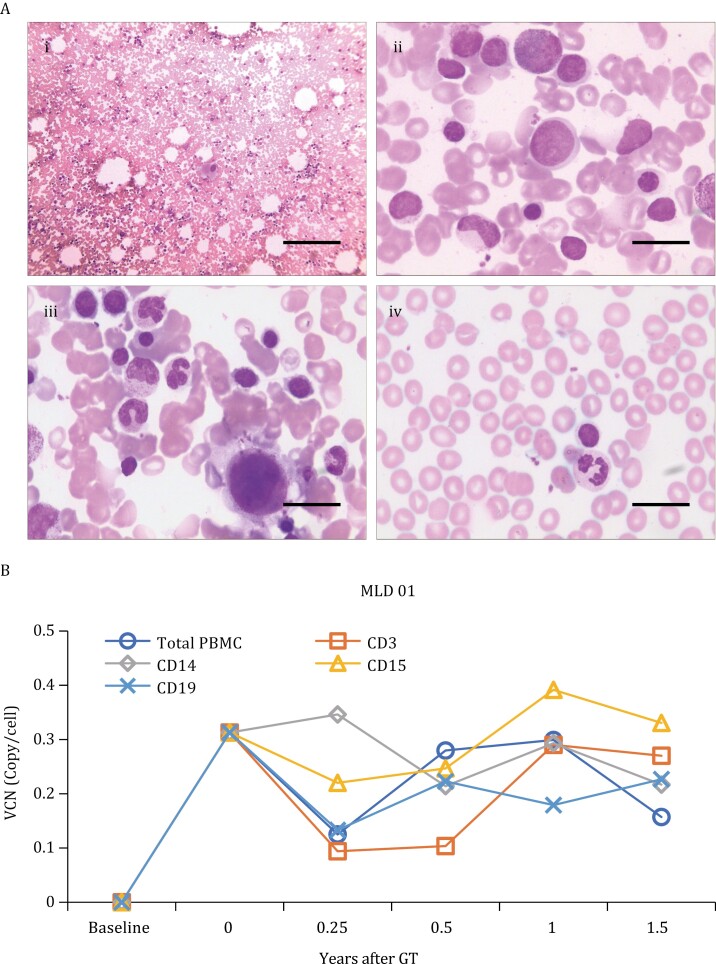
**VCN detection in PBMCs and subpopulations.** (A) (i–iii). Romanowsky-stained smears of bone marrow aspirate from MLD01 6 months after HSCGT show normal cellularity and a ratio (2.1:1) of myeloid to erythroid precursors, consistent with trilineage reconstitution. (iv). The peripheral blood smear shows no abnormal NEUT, lymphocytes, erythrocytes or PLT. (B) Lentiviral vector integration efficiency was determined based on VCN detection in PBMCs and subpopulations of patient MLD01, including CD3^+^, CD14^+^, CD15^+^, and CD19^+^ cells. Continuous monitoring up to 1.5 years of follow-up is shown for patient MLD01.

The genome integration efficiency of lentiviral vectors was assessed by determining the vector copy number (VCN) in CD34^+^ after transduction and in peripheral blood mononuclear cells (PBMCs) of the patients. Following lentiviral vector transduction, the VCN of CD34^+^ was 0.21 in MLD01, 0.79 in MLD02, and 0.32 in MLD03 (Table 1). The VCN of PBMCs was 0.16 in MLD01 at 6.5 years follow-up, 0.08 in MLD02 at 5.1 years follow-up, and 0.26 in MLD03 at 3 years follow-up following HSCGT (Table 1). Patient MLD01 also underwent a bone marrow aspiration smear, and no risk of dysplasia was observed (Fig. 3A). In addition, the VCN of PBMC subpopulations including CD3, CD14, CD15, and CD19 was consistently above 0.1 per cell at 0.25, 0.5, 1, and 1.5-years follow-up for MLD01 (Fig. 3B).

**Figure 4.**
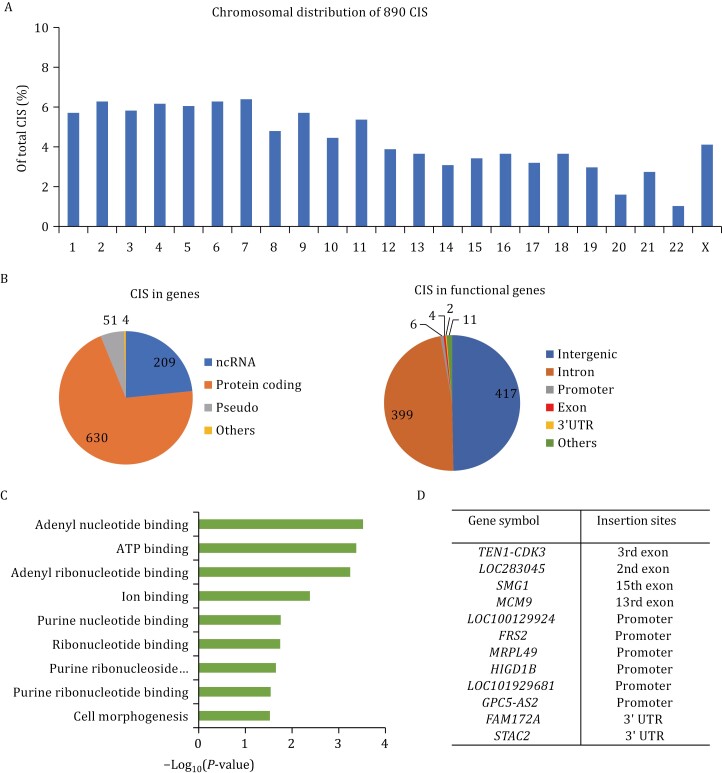
**The lentiviral insertion analysis of MLD01.** (A) Chromosomal distribution of 890 CIS in the blood genome of MLD01 patient. All 890 CIS were evenly distributed among the 23 chromosomes. (B) Most CIS were in intergenic sequences and would not affect gene transcription. Only four CIS were found to be integrated into the exon of functional genes. (C) Gene Ontology of all genes related to 890 CIS. (D) List of 12 functional genes that may be directly affected by 890 CIS.

Of particular concern were the risks of mutagenesis and oncogene activation due to random lentiviral vector insertions. Patient MLD01 was subjected to the genome-wide analysis of lentiviral vector insertions by linear amplification-mediated polymerase chain reaction (LAM-PCR). Chromosomal distribution of 890 chromosomal insertion sites (CIS) was detected in the blood genome of the MLD01 patient. All 890 CIS were evenly distributed among the 23 chromosomes (Fig. 4A). Most CIS were in intergenic sequences and would not affect gene transcription (Fig. 4B). No insertion sites were found in the known oncogenes. Only 12 chromosomal insertion sites were detected in exon, promoter, or 3ʹ UTR regions that could potentially affect gene expression (Fig. 4C and 4D).

### Efficacy assessments

**Figure 5.**
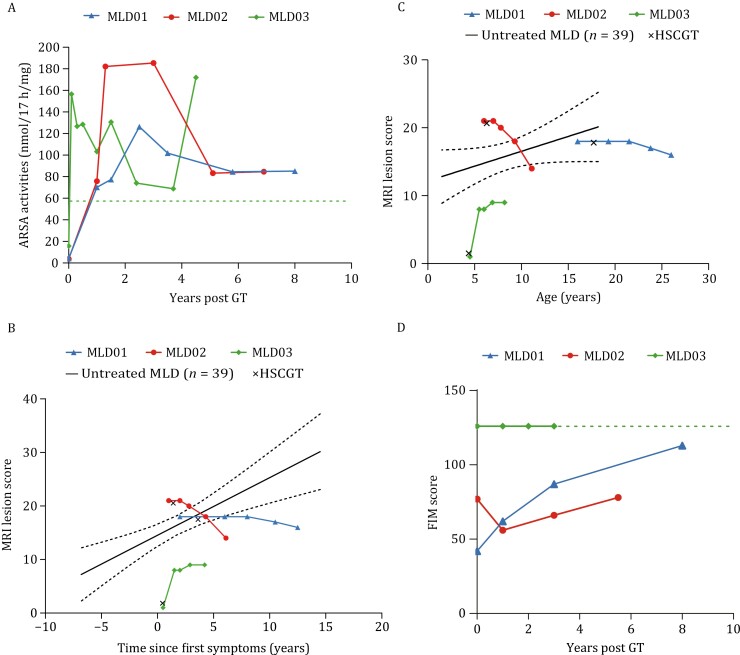
**The natural history, ARSA activity, and FIM scores of MLD patients.** (A) ARSA activity was detected in PBMCs from MLD patients. (B and C) MRI lesion score (median and 95% CI, *n* = 39) of patients with juvenile MLD in relation to time since first symptoms (*P* < 0.001) and age (*P* = 0.08). Several presymptomatic juvenile cases showed MRI lesions prior to symptom onset. The mean of 42 MRIs from 39 untreated patients is shown as a black line. Those for MLD01, MLD02, and MLD03 showed follow-up values below the mean of natural history patients. Lesion severity was assessed using the MRI scoring system for MLD patients. *X* represents a time of HSCGT treatment. (D) FIM score was used to assess the functional impact of neurodegeneration on daily independence in MLD patients. The green dotted line represents the lower limit of the normal range of ARSA activity in healthy individuals.

The secondary endpoints for efficacy assessments included ARSA activity, magnetic resonance imaging (MRI) lesion score, gross motor function classification in MLD (GMFC-MLD), and Functional Independence Measure (FIM) score (Boucher et al., 2015; Fiedler and Granger, 1996; Groeschel et al., 2016; Ottenbacher et al., 1996; Ulaşlı et al., 2014). The serum ARSA activity was significantly improved and consistently sustained above the normal level in all treated patients (Fig. 5A and Table 1).

Linear least squares regression analysis was performed for the juvenile form of the disease to estimate the mean change in MRI lesion scores over time and its 95% CI to examine its relationship with disease course, age, and GMFC-MLD level. Similar to previous reports, the increased MRI lesion score was associated with increased disease duration from the time of first symptoms (*P* < 0.001), age (*P* = 0.08), and GMFC-MLD level (*P* < 0.001) (Figs. 5B and 5C, and S1A) in juvenile MLD patients with a median age of onset at 5.5 years (range 2.5–15.2) (*n* = 39) (Table S2) (Beschle et al., 2020; Groeschel et al., 2016; Krägeloh-Mann et al., 2013). In contrast, in treated patients with a median age of onset at 5.5 years (range 4.0–14.0) (Table 1), the trend in MRI lesion scores in HSCGT patients differed from that in untreated patients (Fig. 5B and 5C). The MRI lesion score in treated patients (mean = 13.00) at the last follow-up was lower than that in untreated MLD patients and increased with disease duration in the natural history cohort (mean = 16.5, 95% CI 12.5–16.6). In our study, MRI lesion scores decreased in patients MLD01 and MLD02 with improved ARSA activity following HSCGT. In addition, a stable disease state was indicated based on the evaluation of GMFC-MLD and FIM scores, suggesting that brain lesions did not deteriorate aggressively with increasing age during long-term follow-up. Although the rapid degeneration of white matter was observed in patient MLD03 within the first 2 years of disease onset, the brain lesions started to stabilize after 1 year of HSCGT and were maintained with a low MRI lesion score over the subsequent 4 years (Table 1; Fig. 5B and 5C).

A significant correlation was found for GMFC-MLD with MRI lesion scores in patients with juvenile MLD (Fig. S1A). Gross motor function was classified based on GMFC-MLD from level 0 (normal) to 6 (loss of any locomotion) (Fig. S1B) (Groeschel et al., 2016). The GMFC level of patient MLD01 improved significantly from level 4 to 0. Gross motor function of patients MLD02 and MLD03 was normal at baseline. Patient MLD03 maintained normal gross motor function, while patient MLD02 was able to walk independently with an unsteady gait at the last follow-up, determined as level 1. In line with the GMFC level, the FIM score improved significantly from maximum assistance to moderate independence in patient MLD01 (Fig. 5D and Table 1) and remained stable in patients MLD02 (moderate assistance) and MLD03 (complete independence).

## Discussion

Once advanced symptoms have developed, there is no cure for patients with MLD. Previous clinical trials have shown that allogeneic HSCT has very limited benefit (Biffi et al., 2013; Sessa et al., 2016). The lack of a suitable HLA-matched donor and the risk of GVHD further limit the chances of patients with MLD to receive timely HSCT (Boucher et al., 2015; Groeschel et al., 2016). Intrathecal delivery of rhARSA has been used, but results have been unfavorable with 25.0% of patients experiencing serious adverse events (SAEs) (Dali et al., 2021). Intracerebral injection of viral vectors or genetically modified HSCs may penetrate the blood–brain barrier, but the invasive surgical procedure and poor biodistribution in the brain limit its application (Capotondo et al., 2017; Sevin et al., 2018). In addition, no clinical benefit has been observed with other targeting molecules involved in sphingolipid biosynthesis, such as warfarin, a vitamin K antagonist (Assadi et al., 2012).

In 2013, Biffi and colleagues first reported a gene therapy strategy based on autologous HSCGT for patients with late infantile MLD in the presymptomatic stage (Biffi et al., 2013; Sessa et al., 2016). No symptoms of neurodegeneration were observed during follow-up prior to the onset of symptoms, while significant disease progression was observed in their siblings in the control group. They suggested that most symptomatic patients would be considered ineligible for treatment due to the rapid disease progression. Recently, the Food and Drug Administration (FDA) has approved the clinical use of HSCGT in children with pre or very early symptomatic MLD. Unfortunately, by the time MLD is diagnosed, most patients have progressed to more advanced symptom onset. Evaluation of the safety and efficacy of HSCGT in this real-world population is urgently needed. Compared with patients who died within a few years after the early-onset form of infantile MLD, those with a late-onset form of juvenile MLD usually show slower neurological progression. This observation provides ample opportunity for potential therapeutic intervention. In the current study with long-term follow-up, patients appeared to benefit from HSCGT treatments, evidenced by significantly improved ARSA activity, and improved GMFC-MLD level and FIM score following treatment, consistent with lower MRI lesion score compared with untreated patients in the natural history cohort (Fig. 5B and 5C).

Regarding the short-term safety assessments within two months of HSCGT, the most common AEs were related to busulfan conditioning and could be controlled by routine treatments. Consistent with a previous report (Sessa et al., 2016), severe neutropenia was reported in all patients in the current study (Table S1). Based on the short-term safety assessments, close monitoring of hematological and hepatocellular enzyme levels is suggested for further studies, as well as monitoring and treatment of busulfan-related AEs and infections. No SAEs occurred during long-term follow-up. Safety data were also favorable for all patients followed for 4.5, 6.9, and 9.6 years, respectively.

The current study has several limitations. First, only three patients at the postsymptomatic stage completed long-term follow-up. Second, MLD is a rare disease with aggressive progression, so recruitment of a large number of patients for a randomized controlled trial and long-term follow-up is difficult (Kehrer et al., 2021). In addition, due to the Severe Acute Respiratory Syndrome Coronavirus 2 pandemic over the last 4 years, gross motor function analysis using the Gross Motor Function Measure-88 (GMFM-88) was not performed. As an alternative, the GMFC-MLD was used, and FIM scoring was used to assess the functional impact of neurodegeneration on daily independence.

Our early pilot study suggests that HSCGT is safe and beneficial for patients with postsymptomatic juvenile MLD. Further studies are needed to investigate its clinical efficacy in a larger sample and to identify biomarkers that predict clinical prognosis.

## Materials and methods

### Study design

From 2014 to 2019, we conducted a multicenter, non-randomized, and open-label clinical trial to evaluate the long-term safety and clinical benefit of HSCGT for children with MLD at the postsymptomatic onset stage at Shenzhen Second People’s Hospital, Shenzhen Children’s Hospital, and Guangzhou Women and Children Medical Center, China (NCT02559830). Ethical approval was obtained from the institutional review boards (IRBs) of all centers before the start of the study (Ethical approval No. XJS2014-001-1, No. 2016-1129, and No. 2017-117). The study was conducted according to good clinical practice guidelines and local health authority regulations. Written informed consent was obtained from all patients and their legal guardians at the time of screening. Data were collected by the investigators and site staff according to the protocol. All patients without a matched HLA donor for allogeneic HSCT and with symptom onset and continuous worsening over 6 months were considered eligible for HSCGT. Detailed methods and natural history of the patients are shown in Figs. 1A, 5B, and 5C, and S2. This report follows the Transparent Reporting of Evaluations with Nonrandomized Designs (TREND) reporting guidelines for nonrandomized controlled trials. Clinical efficacy was assessed individually before and after the investigational treatments. Statistical analysis followed the intention-to-treat principle and was performed using SPSS software (version 20.0, IBM, Armonk, NY, USA).

### Patients

Patients were considered eligible for HSCGT if they fulfilled the following criteria: (a) diagnosis of MLD confirmed by *ARSA* gene mutation; (b) low ARSA activity in peripheral blood (< 20% of the lower limit of normal range); (c) lesions in white matter detected on MRI; (d) symptoms and lesions not progressed to end-stage disease; (e) age < 16.0 years at symptom onset. Patients were excluded if they were any of the following: (a) an adult; (b) at a presymptomatic stage of the disease; (c) at end-stage disease; or if they had (d) ARSA activity in peripheral blood > 50% of the lower limit of normal range; (e) history of malignancy; (f) positive test for hepatitis B, hepatitis C, or human immunodeficiency virus; (g) serious organ dysfunction; (h) had undergone allogeneic HSCT with evidence of residual cells of donor origin; or they (i) were enrolled in other clinical trials in the 6 months prior to screening; or (j) had any other concern that could hamper compliance or safety as judged by the investigator.

### Endpoints

The primary endpoint was the safety of HSCGT as determined by short- and long-term safety assessments following transplantation. Short-term safety was determined by the incidence of treatment-emergent AEs within 72 h of transplantation and tolerance within 2 months of HSCGT. Patients were closely monitored for systemic reactions to the cell infusion for 72 h following HSCT. Potential reactions included fever, tachycardia, nausea, vomiting, arthralgia, and rash. Hematological tests were performed within 2 months of HSCGT to determine hematopoietic reconstitution status. Severe neutropenia was defined as an absolute neutrophil count (ANC) < 500 × 10^6^ neutrophils/L. Graft failure was defined as prolonged hematopoietic reconstitution without evidence of bone marrow recovery. AEs were recorded and monitored for up to 8 years post-transplantation for long-term safety assessment, including but not limited to hematopoietic system disorders. Secondary endpoints included efficacy assessment and exploratory assessments of the integration efficiency of the lentiviral vectors. The efficacy of HSCGT was assessed by improvement in ARSA activity, extent of brain lesions based on MRI score, gross motor function based on GMFC-MLD, and functional impact of neurodegeneration on daily independence according to FIM score. Lentiviral vector integration efficiency was assessed by VCN detection and genomic analysis of lentiviral integration sites.

### Isolation, transduction, and transplantation of CD34^+^ cells

After pretreatment with granulocyte-colony stimulating factor, mononuclear cells were isolated using an apheresis machine, and CD34^+^ cells were further purified using the CD34 Microbead Kit and CliniMACS Prodigy platform (Miltenyi Biotec, Bergisch Gladbach, Germany). The isolated CD34^+^ cells were cultured in HSC expansion media at an appropriate seeding density as verified by flow cytometry and then transduced with a clinical grade lentiviral vector (pCCL.sin.cPPT.hPGK-ARSA.WPRE.LV, MOI: 200) following a protocol modified from a previous publication (Biffi et al., 2013). Replication-competent lentivirus assays were performed by co-culturing transduced CD34^+^ cells with the permissive C8166 cell line. Polymerase chain reaction (PCR) and real-time PCR assays were performed to detect gag-pol mRNA and *VSV-G* DNA 3 weeks after co-culture. All patients received 4 days of chemotherapy to eradicate *ARSA* genetically deficient HSCs prior to HSCGT. Short-term safety monitoring was performed with close hematological monitoring after chemo conditioning with Busulfan (0.8–1.2 mg/kg for 4 days). Then, autologous transfusion of genetically modified HSCs was implemented by the investigators under close monitoring in a special laminar flow unit for bone marrow transplantation in the hematology department which was equipped with electrocardiogram (ECG) monitoring and rescue equipment.

### ARSA activity assay

Cellular ARSA activity was determined using a previously published method (Biffi et al., 2006). Briefly, CD34^+^ cells, PBMCs, and their subpopulations were collected from patients and suspended in sodium acetate trihydrate solution (0.05 mol/L) after several washes to remove the liquid residue. After sonication and centrifugation, the supernatant was collected to measure protein concentration by Bradford assay (Bio-Rad, Hercules, CA, USA) and diluted with sodium acetate trihydrate (0.05 mol/L) to a concentration of 0.3 mg/mL for further assay of ARSA activity. The ARSA activity of the protein extract was assayed using p-Nitrocatechol Sulfate Solution (PNCS, Sigma) as a substrate.

### MRI examination

All patients underwent anatomical MRI on a 1.5 or 3.0 Tesla scanner using a six-channel SENSE head coil (Gyroscan Intera, Philips, Netherlands) MRI scans were performed with the following settings: Spin Echo T1-weighted images, repetition time (TR) 600 ms, echo time (TE) 15 ms, rec matrix 288, 22 slices, 5 and 3 mm thick, axial and sagittal planes; Turbo Spin Echo T2-weighted images, TE120 ms, TR 5500 ms, 24 slices, axial, sagittal, and coronal planes, 4.5 and 3 mm thick; fluid-attenuated inversion recovery Turbo Spin Echo, TE 140 ms, 24 slices, 4.5 mm thick; and volumetric T1 sequence (fast field echo, 110 slices, 1.4 mm thick, and 0 mm gap). Brain lesions were assessed at baseline and post treatment by senior radiology staff blinded to patient treatment using an MRI scoring system (Eichler et al., 2009). The severity of the lesion was categorized as mild (score 1–6), moderate (score 7–15), or severe (score 16–34) according to the total score (Eichler et al., 2009).

### Motor and cognitive scores

GMFC-MLD was assessed using the grading system (Fig. S1B). Motor function was scored from 0 to 6, representing status from normal to loss of any movement (Groeschel et al., 2016). The FIM consists of 18 items to assess motor and cognitive function and has been applied in neurological disorders including MLD in children aged between 6 months and 21 years (Boucher et al., 2015; Fiedler and Granger, 1996; Ottenbacher et al., 1996; Ulaşlı et al., 2014). The scale can be completed by a parent and assesses daily independence to assess the severity of MLD and the effectiveness of treatment. The scale assesses self-care and motor and cognitive abilities that are critical to a patient’s quality of life. The questionnaire is designed to calculate a score for self-care, sphincter control, transfers, locomotion, communication, and social cognition with a total possible score of 126 points. Patient daily independence is categorized as one of seven levels according to the score: complete independence, modified independence, supervision required, minimal assistance, moderate assistance, maximal assistance, and total assistance. A higher score indicates greater independence.

### Vector copy number

The VCN was adjusted for the number of PBMCs (Ma and Chung, 2014). Total genomic DNA was extracted from patient PBMCs using a QIAamp DNA Blood Kit (Qiagen, Hilden, Germany). Plasmids containing long terminal repeat (LTR) and Gastrin releasing peptide (GRP) nucleotide sequences were constructed to generate standard curves for the calculation of VCN using TB Green Premix Ex Taq II (Tli RNase H Plus, Takara Bio, Tokyo, Japan).

### Analysis of CIS

LAM-PCR was used to detect lentiviral vector integration sites as previously reported (Schmidt et al., 2007). Briefly, total genomic DNA was extracted from the patient’s blood cells using a QIAamp DNA Blood Kit (Qiagen, Hilden, Germany). Biotin-modified primers were designed to target the LTR sequences of the lentiviral vector and amplify its flanking human genomic sequence. After the PCR products were captured on beads, the double-stranded DNA was synthesized to construct adapter-mediated DNA libraries. These libraries were further amplified for MiSeq-PE300 sequencing (Centre for Genomic Science, HKU, HKSAR).

## Supplementary data

Supplementary data is available at https://doi.org/10.1093/procel/pwae037.

pwae037_suppl_Supplementary_Materials

## Data Availability

All data are presented in the Figures. All requests for source data are reviewed by the corresponding authors and the clinical data management departments of the relevant hospitals and institutes to determine whether the request is subject to intellectual property or confidentiality obligations. Data will be made available upon reasonable request, except for patient identification information, which is confidential.
