# Supplementary material for: Lentivirus-modified hematopoietic stem cell gene therapy for advanced symptomatic juvenile metachromatic leukodystrophy: a long-term follow-up pilot study
Source: Protein Cell. 2024 Jun 25;16(1):16–27. doi: 10.1093/procel/pwae037 (PMC11700595; doi:10.1093/procel/pwae037)
Supplement: pwae037_suppl_Supplementary_Materials [file pwae037_suppl_supplementary_materials.pdf]

Fig. S1. MRI lesion score in relation to the score for motor deterioration (GMFC-MLD). (A) MRI lesion score of patients with juvenile MLD (n=39) was significantly correlated with the score for motor deterioration (GMFC-MLD) (rho=0.543, $p < 0.001$). MRI lesion scores for patients with motor symptoms (GMFC-MLD level 1-6, $p=0.1 > 0.05$) and those without dyskinesia (GMFC-MLD level 0, $p=0.189 > 0.05$) showed a normal distribution using the Shapiro-Wilk test. An independent sample T-test showed significant differences between these two groups ($p=0.001$). The MRI lesion score of the GMFC-MLD level 0 group (mean score: 12.7; maximum score: 24; minimum score: 0) was significantly lower than that of the GMFC-MLD level 1-6 group (mean score: 19.3; maximum score: 31; minimum score: 13). **(B)** Grading of motor function classification system (GMFC-MLD).

Fig. S2. Genetic diagnosis of MLD patients. c.251G>A and c.439delA mutations were found in MLD01, c.257G>A and c.827C>T mutations in MLD02, and c.925G>A and c.1237G>A mutations in MLD03.

Supplementary Figure 1

A

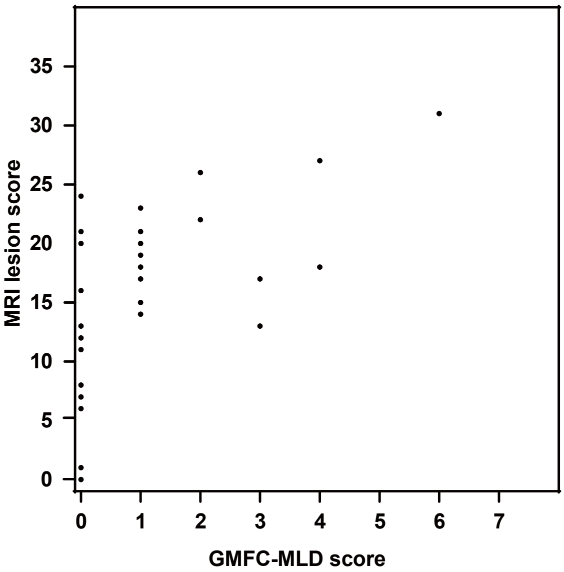

B

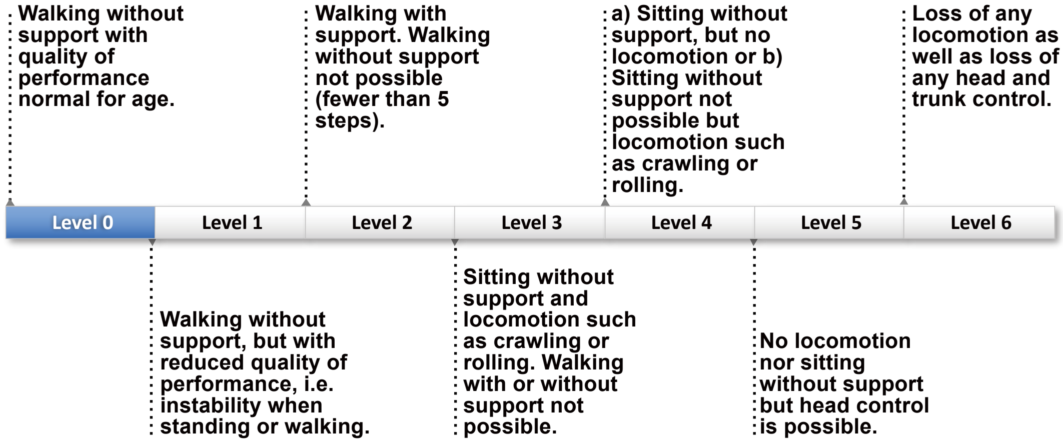

Supplementary Figure 2

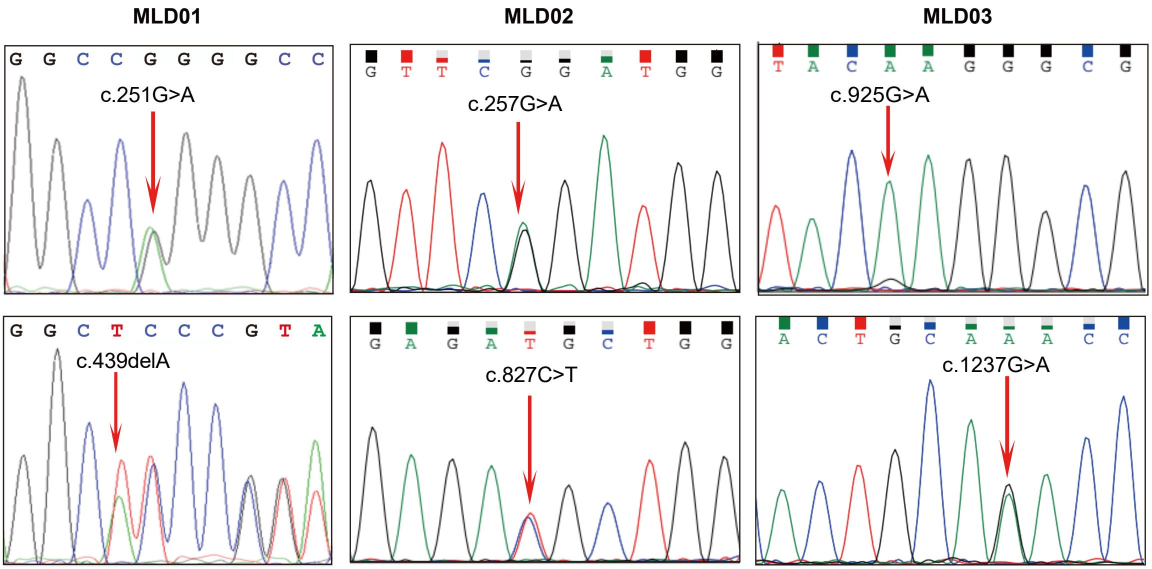

Table S1. Summary of adverse events during short-term safety follow up.

Adverse event	Cases	Case distribution				Duration, mean ±SD, d	Severity			Relative investigational treatment or condition	Outcome
		Conditioning	3d[a]	7d[a]	60d[a]		Mild	Moderate	Severe		
Fever	6	1	-	4	1	3.8 ±1.9	1	1	1	Busulfan conditioning; infection	Recovered
Diarrhea	3		2	-	1	5.7 ±4.5	2	-	-	Drug-induced gastrointestinal disorders	Recovered
Neutropenia	3	-		2	1	5.0 ±2.0	-	-	2	Busulfan conditioning	Recovered
Oral mucositis	3	-	1	1	1	9.0 ±2.6	1	-	1	Busulfan conditioning; infection	Recovered
Vomiting	3	2	-	-	1	4.0 ±2.0	2	-	-	Busulfan conditioning; infection	Recovered
Nausea	2	2	-	-	-	5.5 ±3.5	1	-	-	Busulfan conditioning	Recovered
Epilepsy	1	1	-	-	-	1.0	1	-	-	Busulfan conditioning	Recovered
Convulsion	1	1	-	-	-	1.0	-	1	-	Busulfan conditioning	Recovered
Pancytopenia	1	1	-	-	-	22.0	-	-	1	Busulfan conditioning	Recovered

Adverse events were calculated based on the onsets date within the timeframe following HSCGT.

Abbreviations: HSCGT, hematopoietic stem cell gene therapy; SD, standard deviation.

Table S2. Details of Juvenile Patient Characteristics.

ID	Age at onset (years)	Time since first symptoms (years)	MRI score	GMFC-MLD	Follow-up/ Reference[a]
MLD01	14	2	18	4	Follow-up
MLD02	5.5	1	21	0	Follow-up
MLD03	3.8	0.5	1	0	Follow-up
MLD04	3.3	0.2	6	0	Follow-up
MLD05	9	3	16	0	Follow-up
MLD06	2.7	0.3	19	1	Follow-up
		1	21	1	
MLD07	6.8	0.2	16	0	Follow-up
		0.9	21	0	
MLD8	8	0.3	21	1	Follow-up
MLD9	5.5	3	27	4	Follow-up
MLD10	5	2.7	15	1	Krägeloh-Mann *et al.*, 2013
		14.5	31	6	
MLD11	4.8	0	14	1	Krägeloh-Mann *et al.*, 2013
MLD12	4.8	0.1	14	1	Groeschel *et al.*, 2016
MLD13	4.3	1.7	17	1	Groeschel *et al.*, 2016
MLD14	8.1	2	20	1	Groeschel *et al.*, 2016
MLD15	3.5	7.6	13	3	Groeschel *et al.*, 2016
MLD16	15.2	0	11	0	Groeschel *et al.*, 2016
MLD17	9.4	5.4	8	0	Groeschel *et al.*, 2016
MLD18	7	7.5	17	3	Groeschel *et al.*, 2016
MLD19	10	3	20	0	Groeschel *et al.*, 2016
MLD20	5.3	-0.9	12	0	Groeschel *et al.*, 2016
MLD21	0.3	-0.9	13	0	Groeschel *et al.*, 2016
MLD22	3.7	0.1	19	1	Groeschel *et al.*, 2016
MLD23	5.4	0.4	26	2	Groeschel *et al.*, 2016
MLD24	10	3.2	24	0	Groeschel *et al.*, 2016
MLD25	3.5	3.5	23	1	Groeschel *et al.*, 2016
MLD26	4.6	0	7	0	Groeschel *et al.*, 2016
MLD27	9.4	-0.6	16	0	Groeschel *et al.*, 2016
MLD28	13.8	-6.8	0	0	Groeschel *et al.*, 2016
MLD29	2.5	2.3	22	2	Groeschel *et al.*, 2016
MLD30	5.4	-3.9	0	0	Groeschel *et al.*, 2016
MLD31	13.1	0.8	20	1	Beschle *et al.*, 2020
MLD32	4.3	1.7	17	1	Beschle *et al.*, 2020
MLD33	7.3	4.5	18	1	Beschle *et al.*, 2020
MLD34	9.8	1.8	16	0	Beschle *et al.*, 2020
MLD35	4.8	0.1	14	1	Beschle *et al.*, 2020
MLD36	4.3	0.9	19	1	Beschle *et al.*, 2020
MLD37	9.5	8.7	21	0	Beschle *et al.*, 2020
MLD38	11.4	2.2	18	1	Beschle *et al.*, 2020
MLD39	8.1	2	20	1	Beschle *et al.*, 2020

[a] Patients previously described elsewhere.
